# Comprehensive detection and identification of bacterial DNA in the blood of patients with sepsis and healthy volunteers using next-generation sequencing method - the observation of DNAemia

**DOI:** 10.1007/s10096-016-2805-7

**Published:** 2016-10-22

**Authors:** T. Gosiewski, A. H. Ludwig-Galezowska, K. Huminska, A. Sroka-Oleksiak, P. Radkowski, D. Salamon, J. Wojciechowicz, M. Kus-Slowinska, M. Bulanda, P. P. Wolkow

**Affiliations:** 1 Department of Microbiology, Jagiellonian University Medical College, Krakow, Poland; 2 Center for Medical Genomics OMICRON, Jagiellonian University Medical College, 7C Kopernika Str., 31-034 Krakow, Poland; 3 Genomic Laboratory, DNA Research Center, Poznan, Poland; 4 Laboratory of High Throughput Technologies, Institute of Molecular Biology and Biotechnology, Faculty of Biology, Adam Mickiewicz University, Poznan, Poland

## Abstract

Blood is considered to be a sterile microenvironment, in which bacteria appear only periodically. Previously used methods allowed only for the detection of either viable bacteria with low sensitivity or selected species of bacteria. The Next-Generation Sequencing method (NGS) enables the identification of all bacteria in the sample with their taxonomic classification. We used NGS for the analysis of blood samples from healthy volunteers (*n* = 23) and patients with sepsis (*n* = 62) to check whether any bacterial DNA exists in the blood of healthy people and to identify bacterial taxonomic profile in the blood of septic patients. The presence of bacterial DNA was found both in septic and healthy subjects; however, bacterial diversity was significantly different (*P* = 0.002) between the studied groups. Among healthy volunteers, a significant predominance of anaerobic bacteria (76.2 %), of which most were bacteria of the order *Bifidobacteriales* (73.0 %), was observed. In sepsis, the majority of detected taxa belonged to aerobic or microaerophilic microorganisms (75.1 %). The most striking difference was seen in the case of *Actinobacteria* phyla, the abundance of which was decreased in sepsis (*P* < 0.001) and *Proteobacteria* phyla which was decreased in the healthy volunteers (*P* < 0.001). Our research shows that bacterial DNA can be detected in the blood of healthy people and that its taxonomic composition is different from the one seen in septic patients. Detection of bacterial DNA in the blood of healthy people may suggest that bacteria continuously translocate into the blood, but not always cause sepsis; this observation can be called DNAemia.

**Electronic supplementary material:**

The online version of this article (doi:10.1007/s10096-016-2805-7) contains supplementary material, which is available to authorized users.

## Introduction

The human body is naturally colonized by bacteria, viruses and fungi which occur in specific locations such as the gastrointestinal tract, skin and vagina. However, according to the state of knowledge based on microbial culture, rather than next generation sequencing, most areas inside of the body in healthy man are physiologically sterile [1]. An example of such a sterile microenvironment would be blood, in which bacteria (microorganisms) appear only periodically, e.g. during sepsis. Some researchers have suggested that perhaps in the blood of healthy people traces of bacteria can be found [2]. An example would be asymptomatic bacteraemia, which sometimes occurs as a result of dental procedures, e.g. tooth extraction or orthodontic procedures [3, 4]. However, it is generally agreed that bacteria must be rapidly eliminated from the bloodstream.

The gold standard to diagnose bacteraemia is blood culture using special media, preferably in automatic culture systems, e.g. BACTEC (BectonDickinson). The advantages of this method are the ease of use and low cost of an examination. The disadvantages are its duration, spanning even up to 5 days (until issue of the results) and suboptimal sensitivity, which causes only 15–20 % cultures to have a positive microbial growth [5, 6]. In addition, blood culture method detects only viable bacterial cells. There are also few molecular, culture-independent methods, such as PCR or FISH (Fluorescent In Situ Hybridization), that enable the detection of selected species of bacteria based on the presence of their DNA [7, 8]. These techniques are very sensitive and allow for quick detection of even a very low number of microorganisms in the samples. They have also been commercially adopted to create diagnostic kits, e.g. SeptiFast (Roche), SeptiTest (Molzym), or VYOO (SIRS-Lab); however, they enable detection of only selected species or groups of microorganisms [7]. None of the above-mentioned methods allows to assess the frequency and taxonomical diversity of bacteraemia.

More recently, Next-Generation Sequencing (NGS), which enables the identification of all species of bacteria with their taxonomic classification, was introduced. This method has been successfully applied to a detailed analysis of the human and animal microbiome as well as environmental samples, e.g. soil and seawater [9–11]. Since this method allows to obtain knowledge about all bacteria present in the sample, we can ask: (1) what bacteria exist in the blood of patients with clinical symptoms of SIRS (systemic inflammatory response syndrome), which have not been detected by available diagnostic methods and (2) whether any bacterial DNA is present in the blood of healthy volunteers. There has been no description of the tests carried out using NGS for the analysis of blood from healthy volunteers and sepsis patients yet.

In this study, we describe the results of the application of NGS for the analysis of blood samples from healthy volunteers, compared to patients with clinical symptoms of sepsis.

## Materials and methods

### Samples

Eighty five blood samples were included in this study. Sixty two were from patients hospitalized in the John Paul II Hospital in Krakow at The Ward of Anesthesiology and Intensive Care. They underwent serious cardiac surgical procedures and had clinical symptoms of sepsis according to criteria of the Society of Critical Care Medicine (SCCM) and The European Society of Intensive Care Medicine (ESICM) [12]. The median age of these patients was 67 years. Among them 14 were female and 48 were male. Twenty three blood samples were collected from healthy volunteers who had no clinical symptoms of sepsis and no elevated level of inflammatory markers (CRP, OB). The median age of healthy subjects was 59 years. Among them 13 were female and 10 were male. No patient or volunteer was treated with antibiotics before the collection of blood samples. Blood samples were drawn into 4-ml Vacutainer K3E (BectonDickinson) test tubes.

### Sample analysis

Each of the samples underwent two methods of analysis—microbiological culture and NGS.

### Microbiological culture

Routine blood culture was carried out in the John Paul II Hospital in Krakow in the Microbiology Department using BacT/ALERT® 3D apparatus (bioMérieux).

### DNA isolation, quantitation and quantification

Microbial DNA was isolated from 1.5-ml of blood sample according to the method described by Gosiewski et al. with the employment of a ready-to-use Blood Mini kit (A&A Biotechnology) [13].

The concentration and purity of total DNA isolates in the samples were measured spectrophotometrically (NanoDrop, Thermo Scientific) at wavelengths of A260 and A280.

### 16S Library preparation and sequencing

The library preparation procedure followed the 16S Metagenomic Sequencing Library Preparation Protocol - Preparing 16S Ribosomal RNA Gene Amplicons for the Illumina MiSeq System (Illumina). DNA amplification was performed with the use of KAPA HiFi HotStart ReadyMix kit (KAPA BIOSYSTEMS) on C100 thermal cycler (BioRad), as a nested PCR. The content of PCR reaction mix was the following: 1st amplification—DNA template (2.5 μl), each 1 μM external primers (5.0 μl), KAPA HiFi HotStart ReadyMix (12.5 μl); 2nd amplification—DNA amplicon (2.5 μl), each 1 μM internal primers (5.0 μl), KAPA HiFi HotStart ReadyMix (12.5 μl).

Amplification of hypervariable regions (V3 and V4) of 16S rRNA was used to characterize taxonomic diversity present in blood samples. The universal external primers (Table 1) were designed by aligning to the conservative regions V3 and V4 of 16S rDNA using the procedure described by Gosiewski et al. [14]. A nested amplification was performed using PCR product from the first reaction as a template—the V3 and V4 regions were amplified with region-specific internal primers [15] that included the MiSeq flowcell (Illumina) overhang adapter sequences attached to the 5′ end of primer (Table 1). Further, the 16S library was purified, samples were indexed, amplicon concentrations were quantified and samples were pooled. DNA library concentration was quantified using PicoGreen (Life Technologies).

**Table 1:** Sequences of primers and probes utilized in the study

Amplification	Primer	Sequence 5’–3’	Origin	Target sequences	Amplification program
External	F	ACGGCCNNRACTCCTAC	This study	V3 and V4 16S rRNA	$$ \begin{array}{l}{95}^{\mathrm{O}}\mathrm{C}\hbox{--} 5\ \min \hfill \\ {}{95}^{\mathrm{O}}\mathrm{C}\hbox{--} 15\ \sec \hfill \\ {}\begin{array}{ll}{48}^{\mathrm{O}}\mathrm{C}\hbox{--} 20\ \sec \hfill & 20\times \hfill \end{array}\hfill \\ {}{72}^{\mathrm{O}}\mathrm{C}\hbox{--} 30\ \sec \hfill \\ {}{72}^{\mathrm{O}}\mathrm{C}\hbox{--} 5\ \min \hfill \end{array} $$
	R	TTACGGNNTGGACTACHV			
Internal	F	CCTACGGGNGGCWGCAG	[15]		$$ \begin{array}{l}{95}^{\mathrm{O}}\mathrm{C}\hbox{--} 5\ \min \hfill \\ {}{95}^{\mathrm{O}}\mathrm{C}\hbox{--} 30\ \sec \hfill \\ {}\begin{array}{ll}{55}^{\mathrm{O}}\mathrm{C}\hbox{--} 30\ \sec \hfill & 30\times \hfill \end{array}\hfill \\ {}{72}^{\mathrm{O}}\mathrm{C}\hbox{--} 30\ \sec \hfill \\ {}{72}^{\mathrm{O}}\mathrm{C}\hbox{--} 5\ \min \hfill \end{array} $$
	R	GACTACHVGGGTATCTAATCC			
Overhang adapters^a^	F	TCGTCGGCAGCGTCAGATGTGTATAAGAGACAG	Illumina		
	R	GTCTCGTGGGCTCGGAGATGTGTATAAGAGACAG			

^a^The overhang adapter sequences were added to the internal primer attached to the 5′ end

### Negative control (NTC)

Five samples of sterile water were used as a control of purity of DNA libraries.

### NGS sequencing

The 10pM library containing 90 pooled indexed samples with 20 % spike-in PhiX control DNA was loaded onto the MiSeq (Illumina) apparatus. Sequencing was performed using the MiSeq Reagent Kit v3 (600 cycles). The sequencing procedure was performed in the Genomic Laboratory of the DNA Research Center (Centrum Badań DNA), Poznan, Poland.

### Data analysis and statistics

Sample quality was evaluated using FastQC tool. PCR primers and sequencing adapters were trimmed using the Cutadapt package. Resulting short reads where joined on overlapping regions using the fastq-join tool from the ea-utils package. Both joined and forward unjoined reads were used for further analysis. Reads with base quality lower than 20 were filtered out. OTUs were picked using open-reference protocol. In the first step, closed reference OTU picking is done against the Green Genes 13.08 reference database. The remaining reads that failed to hit the reference database were filtered out and used to perform de novo OTU picking. Reads were clustered using uclust. Taxonomy assignments were performed with PyNAST. Singleton OTUs were removed before further analyses. Relative OTU abundances were calculated using QIIME (www.qiime.org). To estimate alpha diversity chao1, observed OTUs and phylogenetic distance metrics were calculated. Both weighted and unweighted UniFrac distances were calculated to analyze beta diversity, using Student’s t-test. Results were transformed using PCoA and visualized with Emperor. Frequency of OTUs across sample groups was compared using non-parametric ANOVA (Kruskal-Wallis test). Differential abundance of OTUs across sample groups was analyzed using DESeq2 (negative binomial Wald test).

## Results

### Blood culture results

Nineteen out of 62 (30.6 %) blood samples from patients with clinical symptoms of sepsis were positive when analyzed by microbiological culture. In all but three samples from septic patients, the results of blood culture were concordant with the results of next generation sequencing on the genus level. All 23 samples (100 %) collected from volunteers were negative.

### Metagenomic sequencing of the blood microbiome

A total of 4,933,386 reads were generated for the blood microbiome without any unassigned sequences. The minimal number of reads per sample was 59 (maximal 358,089; average 46,133). Samples with less than 10,000 reads (6 out of 62; 9.67 %) were excluded from the analysis.

**Fig. 1:**
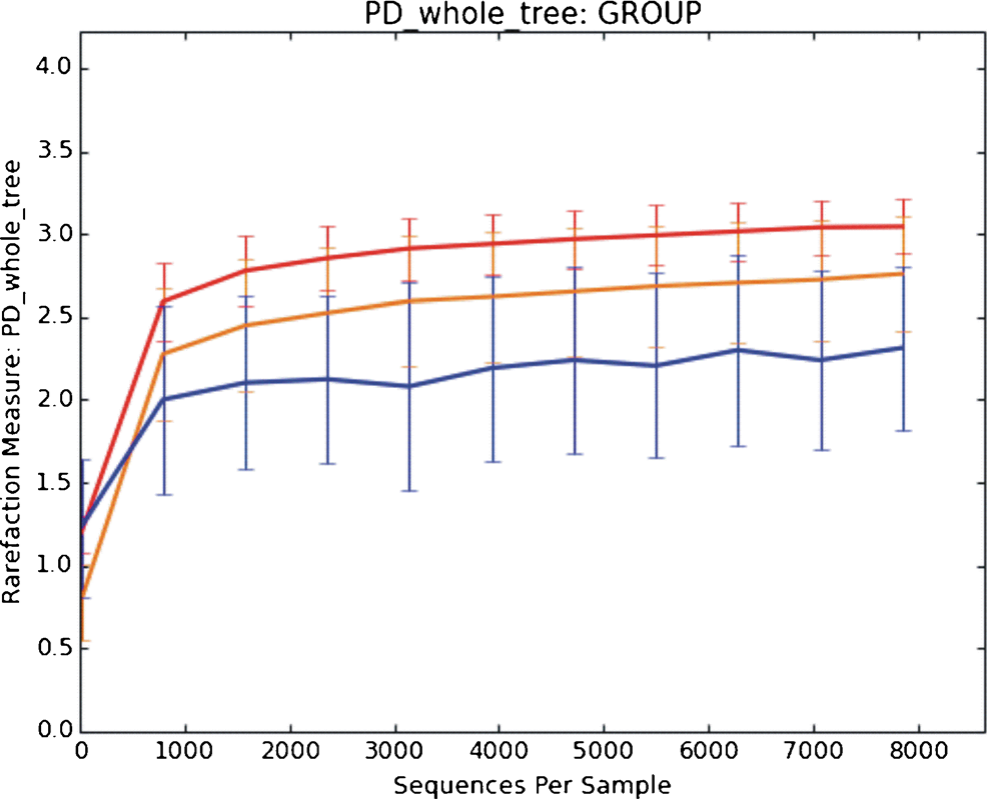
Rarefaction curves for patients with sepsis (*orange curve*) versus healthy patients (*red curve*) and NTC samples (*blue curve*); *p* = 0.002

The presence of bacterial DNA was found in all tested blood samples. Number of reads was comparable between healthy and sepsis; however, bacterial diversity was significantly different (*p* = 0.002) as shown by PD whole tree (Fig. 1), where samples from healthy volunteers were more diverse than sepsis samples. This was not seen when Chao1 or Number of Operational Taxonomic Units metrics were used.

**Fig. 2:**
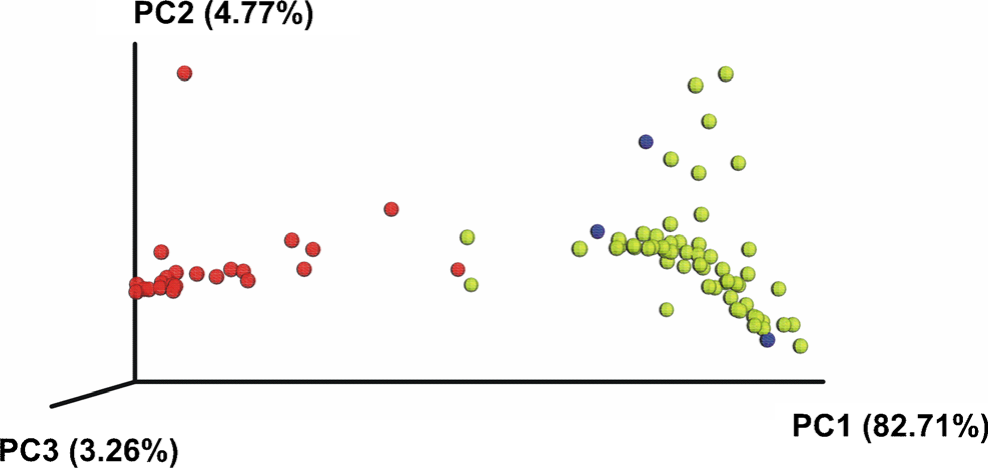
Weighted UniFrac PCoA plot derived from NGS sequencing of blood samples taken from healthy volunteers (*n* = 23, red), NTC samples (*blue*) and patients with sepsis (*n* = 62, *yellow*)

Beta diversity as analyzed both by weighted and unweighted UniFrac, showed significant differences between healthy volunteers and sepsis patients (*P* < 0.001). There was a clear clustering of those who had sepsis and those who were healthy on PCoA (Fig. 2).

**Fig. 3:**
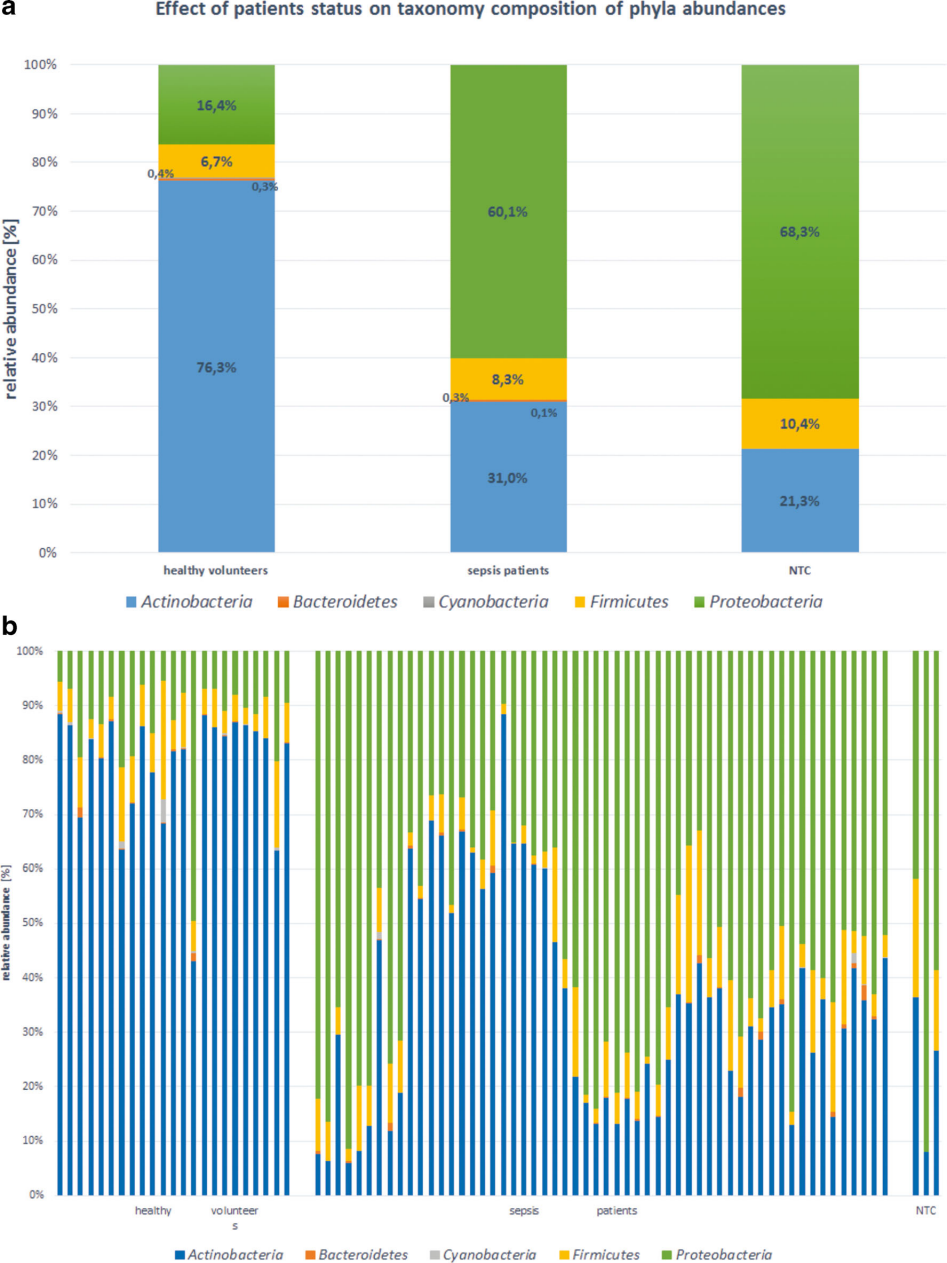
Phyla abundances in the studied groups

In general, differences were observed in the quantitative composition of bacterial taxon between the groups of healthy volunteers and patients with sepsis. *Actinobacteria* phyla abundance was decreased in the sepsis group (from 76.3 % for healthy volunteers to 31.0 % for sepsis group) (*P* < 0.001) (Fig. 3), while *Proteobacteria* phyla abundance was increased (healthy 16.4 %, sepsis 60.1 %; *P* < 0.001) (Fig. 3).

**Fig. 4:**
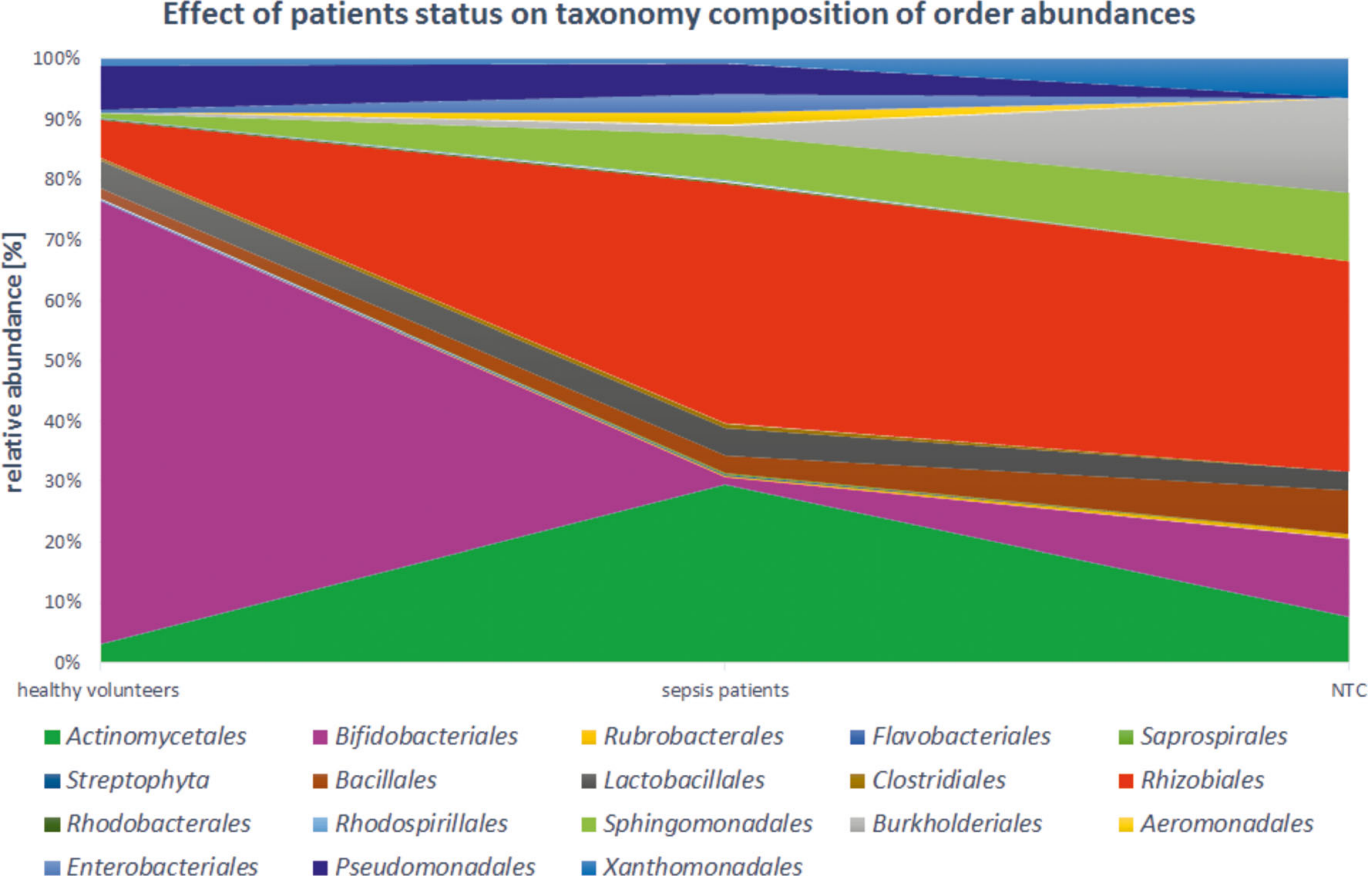
Effect of patients status on taxonomy composition of order abundances

Most sequences from *Proteobacteria* phylum belonged to the following orders: *Pseudomonadales* (7.2 % vs 4.9 %; *P* = 0.01); *Rhizobiales* (6.2 % vs 39.3 %; *P* < 0.001); *Enterobacteriales* (0.2 % vs 3.0 %; *P* = 0.56); *Aeromonadales* (0.1 % vs 2.1 %; *P* < 0.001); *Bacillales* (1.6 % vs 3.0 %; statistically insignificant) and *Sphingomonadales* (0.8 % vs 7.5 %; *P* < 0.001) in the healthy volunteers and in the sepsis group, respectively (Fig. 4). *Actinobacteria* phylum predominantly followed orders *Bifidobacteriales* (73.0 % vs 1.3 %; *P* < 0.001) and *Actinomycetales* (3.2 % vs 29.3 %; *P* < 0.001) in the healthy volunteers and in the septic patients.

In the healthy volunteers, a significant predominance of anaerobic bacteria (76.2 %), of which most were bacteria of the order *Bifidobacteriales* (73.0 %), was observed. In the sepsis group most of the detected taxa belonged to aerobic or microaerophilic microorganisms (75.1 %), i.e. *Pseudomonadales*, *Rhizobiales*, *Enterobacteriales, Aeromonadales, Bacillales* and *Sphingomonadales* and *Cellulosimicrobium* genus (15.3 % vs 0 % in healthy volunteers; *P* < 0.001).

**Fig. 5:**
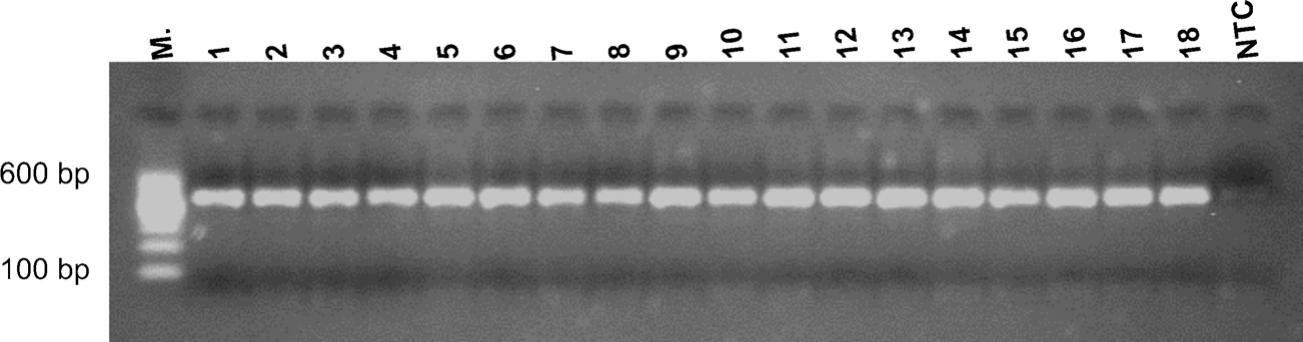
Representative results of nested amplification of the 16S libraries and the negative control (NTC). Amplicon 550 bp

After NTC libraries amplification there was very weak trace of the product on electrophoretic gel (Fig. 5). Of the five NTC samples, three had more than 10,000 reads (the most 54,550 reads). Their quantitative and qualitative taxonomic compositions were completely different from other samples included in the study (see Figs. 3 and 4).

## Discussion

Until recently, molecular biology methods did not allow high-throughput taxonomic classification of bacteria, based on the analysis of their DNA. Development of NGS enabled a thorough investigation of microbial colonization at various body locations. This new technique answers previously unsolved questions, e.g. what kind of bacteria are present in the blood of sepsis patients for whom both culture-dependent and culture-independent methods gave mutually exclusive results or no results at all. Can we find microbial DNA in the blood of healthy people? If the answer to the previous question is positive, which taxonomic unit of bacteria does it come from? As NGS allows to gain a comprehensive profile of bacteria, to the best of our knowledge, we used it for the first time to examine blood samples from patients with clinical signs of sepsis and from healthy volunteers. The NGS method revealed the presence of bacterial DNA in all cases, including blood samples taken from healthy volunteers. However, bacterial diversity was significantly different between healthy and septic subjects (Figs. 1 and 2).

It has been shown by Fitting et al. using PCR method that bacterial DNA can appear in the patients’ blood, even if they have non-infectious SIRS [16]. Similar results obtained using SeptiFast test (Roche) demonstrated the presence of bacteria in 75 % of analyzed blood samples [17]. Gosiewski et al., thanks to the nested PCR method, indicated that 71.8 % of analyzed samples tested positive for bacterial presence [8].

Nikkari et al. indicated the presence of bacterial DNA by PCR in the blood of healthy people [2]. This might be a result of physiological translocation of bacteria from the gastrointestinal tract or the oral cavity or from outside of the body, which however, did not induce sepsis due to the efficient functioning of the immune system [3, 18, 19]. Until now two reports on usage of NGS for detection of bacteraemia in patients’ blood but without symptoms of sepsis were published, namely, after tooth extraction [3] and in AIDS patients [18].

Here, by using NGS, we (1) demonstrate the presence of bacterial DNA in the blood of healthy people and (2) show different quantitative taxonomic composition of bacterial DNA in patients with sepsis compared to healthy volunteers at all taxonomic levels (Figs. 3 and 4). At the level of bacterial phyla we observed a significant decrease in the proportion of *Actinobacteria*, while the percentage of *Proteobacteria* increased in the sepsis group (Fig. 1). Aerobic and microaerophilic bacteria (75.1 %) dominated in patients. Within these, one could see mainly bacteria that often exist in the hospital environment, like *Pseudomonadales*, *Enterobacteriales*, *Bacillales* orders and which are typically known to cause sepsis [20, 21]. Less common were bacteria belonging to the *Sphingomonadales* order and *Cellulosimicrobium* genus (within *Actinomycetales* order), however, in the literature investigators described cases of bacteraemia caused also by these taxa [22–24]. The most surprising was the fact that in the blood of patients with sepsis we detected DNA of *Rhizobiales* order which fix nitrogen and are symbiotic with plant roots. The occurrence of these bacteria could suggest cross-contamination of samples, but there were significant differences between sepsis patients and healthy volunteers (39.3 % vs 6.2 %; *P* < 0.001) which makes explanation by contamination less plausible. Moreover, Lo at al. showed *Rhizobiales* in the blood of patients with fatal pulmonary illness so they can really be causative to the disease [25, 26]. Moreover, no amplification products in the NTC samples can also be used as an evidence for the lack of contamination (Fig. 5). Even though, after sequencing, three samples obtained a sufficient number of reads for further analysis, it was probably the result of amplification during the indexing process. It is known that there are common pollutants of NTC samples that can be detected by NGS. They were listed by Salter et al. [27]. In our study, the most abundant pollutant was *Sphingomonas* genus (7.3 %). According to Laurence et al. the most frequent genus detected in NTC samples was *Bradyrhizobium*, which in our study was indicated at the level of 1.8 % [28].

Blood of the healthy volunteers was dominated by bacterial DNA of the anaerobic *Bifidobacteriales* order (73.0 %) (Fig. 2). Bacteria of this group are part of the human intestinal microbiota and they were reported to have immunomodulatory properties which could potentially prevent infection [29–31]. Shimizu et al. showed that the numbers of total obligate anaerobes and *Bifidobacterium* were severely decreased in patients with major burns and progressed sepsis [32]. Additionally, it was demonstrated in a mouse model, that colonization of both the caecum and the colon by *Bifidobacteria* led to a lesser bacterial contamination of the blood, the liver and the lungs by pathogenic bacteria [33].

In our study we showed a decrease of *Bifibobacteria* and increase of *Proteobacteria* DNA in patients with sepsis, which may be associated with impairment of the intestinal barrier and bacterial translocation [34, 35]. Patients hospitalized in the ICU ward (Intensive Care Unit) in whom the immune system had not worked properly were exposed to nosocomial bacteria, mainly from the hospital environment and from the patient’s own skin microbiota, becoming opportunistic after surgery or other procedures that compromise the protective skin barrier and was reflected in the profile of bacterial DNA (Figs. 1 and 2).

Obtained results show that NGS opens new perspectives in high-throughput microbial diagnostics at many phyla levels. Our research showed that bacteria might continuously translocate into the blood, but not always cause sepsis; this observation can be called DNAemia.

## Electronic supplementary material

Below is the link to the electronic supplementary material.

ESM 1 (PDF 32 kb)
