# Supplementary figures and images for: Comprehensive detection and identification of bacterial DNA in the blood of patients with sepsis and healthy volunteers using next-generation sequencing method - the observation of DNAemia
Source: Eur J Clin Microbiol Infect Dis. 2016 Oct 22;36(2):329–36. doi: 10.1007/s10096-016-2805-7 (PMC5253159; doi:10.1007/s10096-016-2805-7)

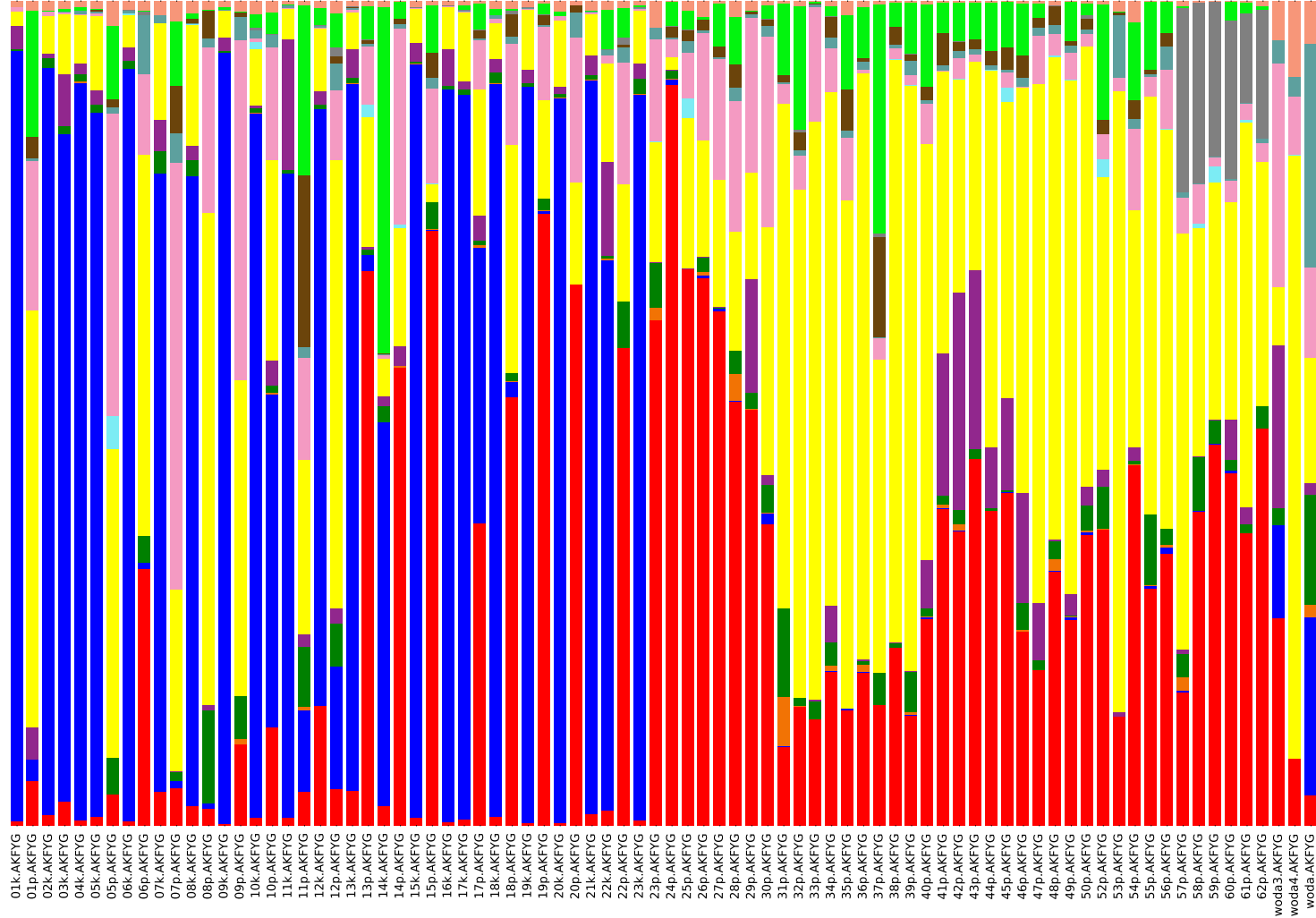

Supplement: Supplementary file 1 — (PDF 32 kb) [file 10096_2016_2805_MOESM1_ESM.pdf]
